# Critical Relevance of Stochastic Effects on Low-Bacterial-Biomass 16S rRNA Gene Analysis

**DOI:** 10.1128/mBio.00258-20

**Published:** 2020-06-09

**Authors:** John R. Erb-Downward, Nicole R. Falkowski, Jennifer C. D’Souza, Lisa M. McCloskey, Roderick A. McDonald, Christopher A. Brown, Kerby Shedden, Robert P. Dickson, Christine M. Freeman, Kathleen A. Stringer, Betsy Foxman, Gary B. Huffnagle, Jeffrey L. Curtis, Sara D. Adar

**Affiliations:** a Pulmonary & Critical Care Medicine Division, Internal Medicine Department, University of Michigan, Ann Arbor, Michigan, USA; b Department of Epidemiology, School of Public Health, University of Michigan, Ann Arbor, Michigan, USA; c Research Service, VA Ann Arbor Healthcare System, Ann Arbor, Michigan, USA; d Center for Molecular Clinical Epidemiology of Infectious Diseases, School of Public Health, University of Michigan, Ann Arbor, Michigan, USA; e Graduate Program in Immunology, University of Michigan, Ann Arbor, Michigan, USA; f Department of Microbiology & Immunology, University of Michigan, Ann Arbor, Michigan, USA; g Medicine Service, VA Ann Arbor Healthcare System, Ann Arbor, Michigan, USA; h Michigan Center for Integrative Research in Critical Care, University of Michigan, Ann Arbor, Michigan, USA; i Department of Clinical Pharmacy, College of Pharmacy, University of Michigan, Ann Arbor, Michigan, USA; University of Maryland School of Medicine

**Keywords:** 16S rRNA gene, contamination, exhaled breath condensate, low biomass, lung microbiome, sequencing noise, next-generation sequencing

## Abstract

The bacterial microbiome of human body sites, previously considered sterile, remains highly controversial because it can be challenging to isolate signal from noise when low-biomass samples are being analyzed. We tested the hypothesis that stochastic sequencing noise, separable from reagent contamination, is generated during sequencing on the Illumina MiSeq platform when DNA input is below a critical threshold. We first purified DNA from serial dilutions of Pseudomonas aeruginosa and from negative controls using three DNA purification kits, quantified input using droplet digital PCR, and then sequenced the 16S rRNA gene in four technical replicates. This process identified reproducible contaminant signal that was separable from an irreproducible stochastic noise, which occurred as bacterial biomass of samples decreased. This approach was then applied to authentic respiratory samples from healthy individuals (*n* = 22) that ranged from high to ultralow bacterial biomass. Using oral rinse, bronchoalveolar lavage (BAL) fluid, and exhaled breath condensate (EBC) samples and matched controls, we were able to demonstrate (i) that stochastic noise dominates sequencing in real-world low-bacterial-biomass samples that contain fewer than 10^4^ copies of the 16S rRNA gene per sample, (ii) that critical examination of the community composition of technical replicates can be used to separate signal from noise, and (iii) that EBC is an irreproducible sampling modality for sampling the microbiome of the lower airways. We anticipate that these results combined with suggested methods for identifying and dealing with noisy communities will facilitate increased reproducibility while simultaneously permitting characterization of potentially important low-biomass communities.

## Abstract (precis)

DNA contamination from external sources (reagents, environment, operator, etc.) has long been assumed to be the main cause of spurious signals that appear under low-bacterial-biomass conditions. Here, we demonstrate that contamination can be separated from another, random signal generated during low-biomass-sample sequencing. This stochastic noise is not reproduced between technical replicates; however, results for any one replicate taken alone could look like a microbial community different from the controls. Using this information, we investigated respiratory samples from healthy humans and determined the narrow range of bacterial biomass where samples transition from producing reproducible microbial sequences to ones dominated by noise. We present a rigorous approach to studies involving low-bacterial-biomass samples to detect this source of noise and provide a framework for deciding if a sample is likely to be dominated by noise. We anticipate that this work will facilitate increased reproducibility in the characterization of potentially important low-biomass communities.

## INTRODUCTION

The advent of next-generation sequencing sparked a keen interest in investigating the microbial communities of sample types from locations or environments that were previously considered sterile or too low in microbial biomass to characterize accurately (1–9). Many of these investigations have fundamentally changed our definition of a sterile site and have demonstrated the large impact that low-bacterial-biomass communities can have, for example, on a human host. However, these studies also have raised critical issues and controversy due to irreproducible results (10–14). The identification of DNA contamination from the laboratory and in reagents used for DNA isolation and sequencing (15) helped greatly in trying to detangle spurious signal that appeared out of nowhere. Nevertheless, results in studies of low-bacterial-biomass samples that cannot be reproduced remain common, despite consideration of proper quality control samples (10, 13, 14). Other studies have considered the effect of barcode or index switching, whereby mistakes in reading the identifier for a sequencing group cause the sequence to be attributed to a different group (16–18). Although this effect has largely been studied on the Illumina HiSeq, the effect in amplicon sequencing does not appear to be large (18). A very recent study identified well-to-well contamination as a major contributor to variation; however, even after repeated assays using multiple thermocyclers, handling robots, and separate locations, no consistency could be found in the variation associated with any given library preparation method (18). This finding suggests that another factor is also at play.

The goal of this study was to test the hypothesis that a stochastic sequencing noise is generated during sequencing on the MiSeq platform—and, likely, other platforms sharing that sequencing design—when sequencing input is below a critical threshold. Stochastic sequencing noise can lead to erroneous and irreproducible conclusions when interpreted as authentic sequences within a given sample. We tested this hypothesis using both laboratory-generated serial dilutions of DNA extracted from Pseudomonas aeruginosa strain PAO1 and authentic respiratory samples collected from healthy human subjects.

For the purposes of this study, we define the “signal” of the sequencer as classifiable sequences shared between technical replicates and “noise” as sequences that can be classified but which lack consistency between technical replicates. In contrast, “contamination,” such as reagent contamination, is defined as a signal shared between replicates that is also present in experimental controls. Thus, contamination can be detected only with the appropriate controls. It is also worth noting, however, that even in cases where controls fail to amplify, the consistency, or lack thereof, between sample replicates still provides critical information as to the balance of signal and noise within the sample.

## RESULTS

### Identifying sequencing noise beyond lab and reagent contamination.

**FIG 1:**
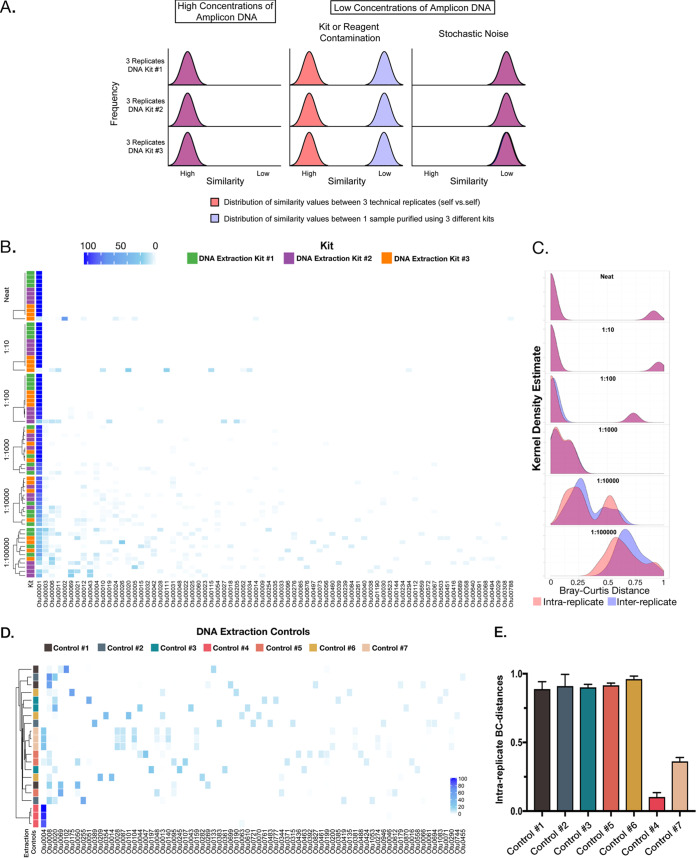
Serial dilutions of P. aeruginosa DNA purified using three separate DNA isolation kits and sequenced in quadruplicate: effects of low biomass on results. (A) An idealized model of contamination effects on a single sample purified using 3 separate DNA isolation kits and 3 technical replicates for each of those kits. Contamination within each kit is assumed to be 100% different from that of reagents in all other kits. The distribution of a hypothetical similarity score is plotted along the *x* axis for similarity between technical replicates (in red) and between kits (in blue). Where overlap occurs, the curves appear purple. The first column depicts the condition of high concentrations of DNA, the second column depicts low concentrations of DNA where kit or reagent contamination is dominant, and the third column depicts low concentrations of DNA where random noise dominates. (B) Heat map of the top 100 OTUs (horizontal axis) broken down by dilution (vertical axis) and grouped using a complete linkage clustering. Note emergence of increasing numbers and diversity of low-abundance reads with increasing dilution. (C) Kernel density estimates for the intrareplicate (within-kit) and interreplicate (between-kit) Bray-Curtis distance for each dilution series. (D) Heat map showing the individual results from reagent controls with technical replicates (*n* = 3/sample). Samples are grouped using a complete linkage clustering. The DNA isolation kit is indicated by the color displayed to the left of the heatmap. (E) Graph depicting the interreplicate Bray-Curtis from technical replicates of reagent controls; bars are colored by kit as in panel D.

We began by modeling how different types of contamination effects could be visualized using the similarity of different kinds of replicates (Fig. 1A). Figure 1A presents an idealized model of contamination effects on a single sample purified using 3 separate DNA isolation kits and 3 technical replicates for each of those kits. Contamination within each kit is assumed to be 100% different from contamination in all other kits. The distribution of a hypothetical similarity score is plotted along the *x* axis for similarity between technical replicates (in red) and between kits (in blue). Where overlap occurs, the curves appear purple. The first column depicts the condition of high concentrations of DNA, the second column depicts low concentrations of DNA where kit or reagent contamination is dominant, and the third column depicts low concentrations of DNA where random noise dominates. The critical takeaway from this modeling is that all specific forms of DNA contamination should result in technical replicates looking highly similar. We tested this by sequencing the 16S rRNA gene from serial dilutions of DNA isolated from a pure bacterial culture of a single bacterial strain, in this case, Pseudomonas aeruginosa PAO1. DNA was isolated using three separate DNA isolation kits, and each dilution was sequenced using four technical replicates. Consistent with previous observations, as the concentration of input DNA decreased, the bulk of the community membership was composed of reads belonging to operational taxonomic units (OTUs) unrelated to P. aeruginosa and thus spurious (Fig. 1B). For example, at a dilution of 1:100,000, only 28.65% ± 4.23% (mean ± standard error of the mean [SEM]) of reads comprised the OTU corresponding to PAO1. To assess the consistency of such noninput sequencing signals, we compared the Bray-Curtis distances for each dilution between isolation kits (i.e., interreplicate distances) and within technical replicates (i.e., intrareplicate). We plotted the results for each dilution set as a density distribution estimate of the intra- and interreplicate Bray-Curtis values (Fig. 1C). Again, in the model where contamination (in this case, kit contamination) is the major driver of sequencing noise, the two peaks should stay together at high concentrations but separate with dilution. Additionally, intrareplicate distance should stay low (technical replicates should look similar). As expected, the distribution of the interreplicate distances (in blue) increased, centering on higher values (i.e., the kits became more dissimilar from each other) as the DNA was diluted. However, contrary to expectation, the intrareplicate distances (in red) also increased as the DNA was diluted and overlapped the interreplicate distances. This finding shows that the replicates from a single kit do not look like themselves, indicating a stochastic source of variation.

Reagent contamination is well documented but was not observed in our initial experiments, so we next tested reagents from seven separate DNA isolation kits by carrying out the DNA isolation process on reagents alone. These controls were sequenced in triplicate, the resulting OTUs were plotted as a heat map (Fig. 1D), and the intrareplicate Bray-Curtis distances were determined (Fig. 1E). Two of the seven controls demonstrated low intrareplicate Bray-Curtis distances and communities consistent with reagent contamination; however, the remaining five controls demonstrated diverse communities that had little or no similarity between technical replicates. Collectively, these data demonstrate that sequencing of dilute samples or reagents can result in a nonreplicable, stochastic, positive signal (sequencing noise) even in the absence of detectable DNA contamination within the control reagents.

### Effect of sequencing noise on samples ranging from high to low bacterial biomass.

We next examined a group of clinical samples from healthy human volunteers (Table 1) to determine how sequencing noise might affect result interpretation. Respiratory samples were chosen because they span the range from high to ultralow bacterial biomass. To that end, we analyzed samples from the following sources: oral rinse, posterior nasopharyngeal swab, bronchoalveolar lavage (BAL) fluid from right (R) and left (L) lungs, a sample containing an equal mix of RBAL and LBAL (CBAL), and an exhaled breath condensate (EBC) sample. EBC is of considerable interest because, as a noninvasive sample, it could greatly expand the type of microbiological questions that could be addressed in epidemiological studies if it accurately reflects lower airway microbial communities. We matched each sample type to the negative controls to which it was best suited.

**TABLE 1:** Demographics and clinical data of human research participants

Sex	Age (yrs)	Race	Pack-yrs	Smoking status	Vol (liters) (% predicted)^*a*^		FEV_1_/FVC
					FEV_1_	FVC	
Female	25	White	0	Never	3.37 (96)	3.84 (95)	0.88
Male	68	White	0	Never	4.96 (109)	6.20 (114)	0.80
Female	61	White	0	Never	2.36 (89)	2.55 (75)	0.93
Female	73	White	0	Never	1.94 (93)	2.21 (84)	0.88
Female	59	White	0	Never	2.24 (94)	2.54 (98)	0.88
Male	22	White	0	Never	4.62 (100)	5.92 (106)	0.78
Female	24	Black	0	Never	2.72 (82)	2.98 (82)	0.91
Male	53	White	0	Never	3.95 (111)	4.17 (94)	0.95
Female	55	Black	20	Former	2.21 (82)	2.82 (84)	0.78
Female	55	Black	20	Former	2.30 (91)	2.51 (91)	0.92
Female	71	White	21	Former	1.72 (85)	1.97 (79)	0.88
Male	58	White	48	Former	3.30 (98)	4.16 (98)	0.79
Male	43	White	22.5	Current	4.41 (119)	5.34 (116)	0.83
Female	59	White	10.5	Current	2.59 (81)	3.00 (86)	0.86
Male	58	White	28.5	Current	3.50 (89)	4.59 (95)	0.76
Male	53	White	10.5	Current	3.02 (83)	3.46 (77)	0.87
Male	38	White	33	Current	4.74 (108)	5.63 (106)	0.84
Male	63	White	35	Current	3.12 (88)	3.83 (87)	0.81
Male	67	Black	25	Current	2.28 (63)	3.56 (80)	0.64
Female	59	White	20	Current	1.38 (53)	2.00 (60)	0.69

a FEV_1_, forced expiratory volume in 1s; FVC, forced vital capacity; Pack-yrs, number of packs smoked per day times number of years smoked.

We extracted DNA from both samples and controls (including experimental controls [e.g., sterile saline passed through the bronchoscope before bronchoscopy] and isolation controls [e.g., purified DNA from DNA isolation reagents]) as described in Materials and Methods, and from this we amplified the V4 region of the bacterial 16S rRNA gene in triplicate using a dual-barcoding strategy and following a low-biomass protocol (19). Finally, we sequenced each sample using an Illumina MiSeq instrument.

**FIG 2:**
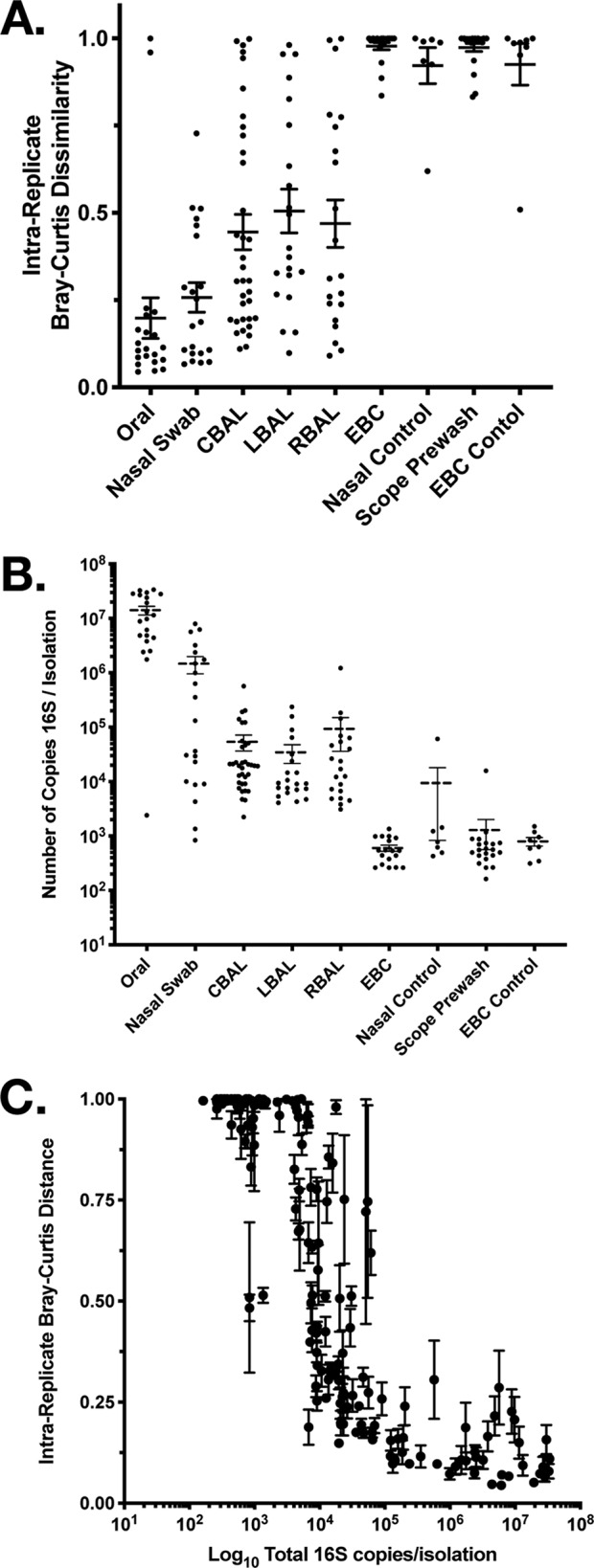
Relationship between the number of 16S rRNA gene copies in a sample and the reproducibility of the result. (A) Intrareplicate Bray-Curtis distance by sample type. Relationship between the number of bacterial 16S rRNA gene copies in a sample and the intrareplicate Bray-Curtis distance between replicates of respiratory specimens and their individual controls. (B) Mean (± SEM) number of 16S rRNA gene copies per sample by sample type. (C) Concentration of the number of bacterial 16S rRNA gene copies in a sample plotted against the intrareplicate Bray-Curtis distance between replicates of respiratory specimens and their individual controls. Each value is the mean ± SEM.

First, we examined whether biological samples were different from their respective controls. In each of these comparisons, the samples were found by permutational multivariate analysis of variance (MANOVA) (adonis) to contain significantly different communities than the controls (oral rinse versus saline, *P* = 0.001; nasal swab versus control swabs, *P* = 0.001; right BAL fluid versus scope prewash, *P* = 0.001; left BAL fluid versus scope prewash, *P* = 0.001; CBAL fluid versus scope prewash, *P* = 0.001; EBC versus EBC control, *P* = 0.003). In the model where the only sources of signal were either genuine or contamination, such results suggest that the signal in every instance should be considered a real community. However, when the similarity or dissimilarity of technical replicates (as measured by intrareplicate Bray-Curtis distance) was examined, it became clear that the communities in certain sample types (e.g., oral) were more reproducible than others (e.g., BAL fluid) and that some (e.g., EBC) were as irreproducible as reagent controls (Fig. 2A). This finding suggested a relationship between the number of 16S rRNA gene copies in each sample (Fig. 2B) and reproducibility. When this was explicitly tested (Fig. 2C), it became clear that not only was there a definite relationship between bacterial biomass and sample reproducibility but also that one can predict whether a sample will need technical replication to separate signal from noise. We found that there exists a very narrow range (10^3^ to 10^4^ 16S rRNA gene copies per sample) within which samples went from being reproducible between technical replicates to instead being irreproducible and dominated by noise. The majority of control samples fell within or below this range (Fig. 2B).

**FIG 3:**
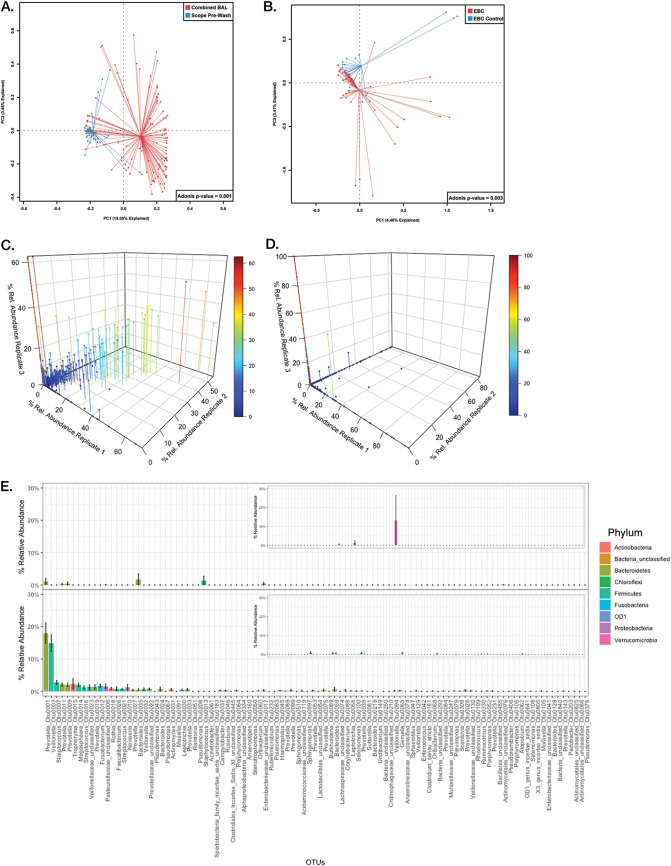
Comparison of the representation of the lung microbiome in EBC versus CBAL. (A) Principal component analysis (PCA) graph depicting CBAL samples (red) and scope prewash controls (blue). (B) PCA graph depicting EBC (red) and EBC controls (blue). (C) 3D scatterplot of CBAL sample OTU abundances, where each replicate is plotted on a separate axis. Common signals between each replicate should appear along the diagonal of the 3D box. Drop lines anchor the points to a position in the *x*-*y* plane, whereas color reflects higher abundances along the *z* axis. (D) 3D scatterplot of EBC sample OTU abundances, where each replicate is plotted on a separate axis. Drop lines anchor the points to a position in the *x*-*y* plane, whereas color reflects higher abundances along the *z* axis. (E) Rank abundance plots of the means of replicate medians of EBC (top) compared to CBAL fluid (bottom). Plots are ordered according to the mean abundances of the CBAL samples. Insets are the sample controls (EBC control and scope prewash control, respectively) ordered by mean abundances of CBAL samples. Bars show means of replicate medians ± SEM and are colored by the phylum of the OTU.

Finally, we applied what we had learned to the question of whether EBC can be used as an accurate replacement for BAL fluid in determination of the lung microbiota. The combined BAL fluid (CBAL) and the EBC were separable from their controls (Fig. 3A and B); traditionally, this result would suffice to consider each to have a detectable microbiome that could be compared. However, as has been shown, the intrareplicate distances of technical replicates suggest that noise can play a large role in these samples. To visualize the effect of this noise on the communities in question, we plotted each replicate set against the other two for both CBAL and EBC (Fig. 3C and D). A sample composed of all specific signal and no noise would be expected to have a perfect correlation between all OTUs detected within each technical replicate (i.e., a diagonal line across a 3D box of the 3 replicates); in contrast, a sample with only noise would have no correlation (i.e., data distributed along the axes of the 3D box). Examination of the CBAL sample (Fig. 3C) shows that, while noise is present, a consistently reproducible signal exists across all the CBAL samples. The same cannot be said for the EBC samples, as very few points appear off the axes (Fig. 3D). In this situation, the standard method of taking the mean of the abundance of community members would be an inaccurate representation of the community due to the preponderance of outliers. A more accurate method to assess the reproducible portion of the community across multiple samples is to take the mean of replicate medians, which deemphasizes the stochastic noise between samples.

We applied this approach to the EBC and CBAL samples and plotted the results according to the average rank abundance of the CBAL samples (Fig. 3E). The EBC samples both lacked consistent community structure and did not resemble the CBAL samples (Fig. 3E, top). In contrast, across all CBAL samples, we reproducibly observed 30 to 40% of the BAL fluid community to be made up of *Prevotella* spp. and *Veillonella* spp., with smaller but real abundances of other community members (Fig. 3E, bottom). This is an important and validating finding, as *Prevotella* spp. and *Veillonella* spp. have been found to be dominant community members in most lung microbiome studies. Neither sample set appeared to be significantly affected by reagent contamination (Fig. 3E, insets).

## DISCUSSION

Collectively, our data analyzing both a defined bacterial community and real human clinical samples demonstrate that (i) stochastic noise occurs in real-world samples of low bacterial biomass; (ii) below a total of 10^4^ copies of 16S rRNA gene in a sample (as determined by droplet digital PCR), one needs to worry about noise dominating real sequences; (iii) critical examination of the composition of technical replicates can be used to separate signal from noise; and (iv) EBC is not a satisfactory sample for sampling lower respiratory bacterial microbiota from healthy individuals using the sequencing method that we tested.

These data extend the now-foundational study by Salter and colleagues (15), which demonstrated that when low-biomass samples are being sequenced, failure to sequence relevant controls simultaneously can cause even the most carefully designed study to be skewed by DNA contamination within reagents. The current study demonstrates that stochastic sequencing noise is an additional critical element to consider when low-biomass samples are being sequenced, as it can produce results that can be easily mistaken for real signal. We show that quantifying 16S rRNA gene copy number before sequencing can determine which samples will be most susceptible to being dominated by noise. To that end, we recommend that any sample containing <10^5^ 16S rRNA gene copies/sample be treated as likely to be significantly affected by noise. Furthermore, because sequencing of technical replicates allows one to set up a necessary baseline (20), replicate sequencing of vulnerable samples can efficiently allow one to differentiate real signal from noise and can aid in detecting contaminants.

Replicate sequencing also introduces an element of precision into an analysis that frequently possesses no true negative controls. Sequencing techniques cannot prove that a sample for microbiome analysis is negative (i.e., contains absolutely no microbial DNA). However, one can say within precision of *x* number of replicates (e.g., three) that no consistent signal was found. Using this approach, which must obviously balance degree of precision versus cost, we concluded that EBC is a poor sample for 16S rRNA gene assessment. We base this conclusion not on an assertion that it was negative or that it did not differ from its controls but on the fact that within the number of replicates that we ran on each sample, we were unable to find a consistent signal. This approach should be applied to other low-biomass sample types that remain controversial, such as peripheral blood and placenta (21).

Our data suggest that the origin of this stochastic noise is related to the sequencer itself, most likely due to underloading of the flow cell and very low cluster densities. While future studies will be needed to uncover the specific problems within the system, it is not hard to imagine a situation where the weak signal produced from very low cluster densities could result in an increase in the sensitivity (gain) of the detectors, causing an increase in detector noise and cross talk between channels. Likely added to these problems is the sum of many sample-side low-frequency events, such as PCR error (22), index switching (17), and chimera formation (22), each of which likely is magnified in the absence of a strong signal. Indeed, while this study employed state-of-the-art methods for error reduction for sample processing, it seems very likely that the error associated with the above-mentioned factors is different, and likely higher, under low-biomass conditions (23). However, we do not believe that differences in these sources of error alone can explain the extent of the irreproducible differences seen in the low-biomass context. Changes in the overall error rate could be monitored by following replicate dilutions of a mock community, but this cannot separate the factors contributing to the errors. Changes in the frequency of index switching could potentially be monitored by using a combination of sample replication using standard dual indexing barcodes compared to the unique dual index barcodes (17). A recent study identified well-to-well contamination as a likely contributor to the unexpected sequencing results (18); however, the extent of the effect varied greatly depending on library preparation method, run, and location, so it is unlikely that well-to-well crossover would be the only noncontamination cause of sequencing noise. Regardless of the cause, the methods for identifying and dealing with noise described here should improve reproducibility. An interesting corollary to the idea that the noise is associated with the sequencer is that running the same samples on a sequencing platform based on a different underlying technology, such as Oxford Nanopore Technologies MinION, would result in a completely different behavior from sequencing controls and low-biomass amplicons. In summary, low-biomass sequencing experiments push the lower limits of sequencers’ capacity to detect signal accurately, so it should be unsurprising that they exceed the threshold of noise that exists in every detection system.

### Conclusions.

We have identified a stochastic sequencing noise which occurs in low-biomass samples and which is experimentally separable from reagent contamination. Additionally, we identified the range of bacterial DNA concentrations beyond which technical replicates, although expensive, are likely to aid in separating signal from noise and hence improve confidence in results. Finally, we tested these methods on respiratory samples and used our knowledge of sequencing noise to inform our decision that EBC is a poor sample to assess the lung microbiota.

## MATERIALS AND METHODS

### Pseudomonas aeruginosa DNA dilutions.

We obtained cells for isolation of bacterial DNA by growing P. aeruginosa strain PAO1 in a 125-ml disposable plastic Erlenmeyer flask containing 20 ml LB broth, which was agitated overnight on an orbital shaker at 125 rpm in a 37°C incubator. Three 1-ml aliquots, collected in 2.0-ml dolphin-nose microcentrifuge tubes, were spun down in a microcentrifuge at a relative centrifugal force (rcf) of 18,000 at 4°C for 30 min, after which the supernatant was discarded. Each tube was then subjected to DNA extraction using a separate DNeasy kit from Qiagen (MD, USA) according to manufacturer protocols with minor modifications (19) and with the addition, after resuspension of the bacterial pellet in lysis buffer, of a 30-s bead-beating step (using garnet beads [Qiagen, MD, USA]). Once DNA was obtained (referred to as neat), it underwent five serial dilutions (1:10 each) in kit elution buffer. For sequencing, each of the samples from each of the dilutions was sequenced in quadruplicate.

### Clinical samples and controls.

We recruited 20 healthy volunteers to provide EBC, oral wash, posterior nasopharyngeal swab, and BAL fluid samples for microbial analysis. Participants had a mean age of 53 ± 15 years, were 50% female, were 20% black and 80% white, and were all of non-Hispanic ethnicity (Table 1). They were a mixture of persons who had never smoked, former smokers, and current smokers. With the exception of two participants with undiagnosed moderate airflow obstruction, they were without evidence of lung disease, and none was taking any inhaled medications. Prior to bronchoscopy, all participants underwent a complete history and physical examination by a pulmonologist, spirometry, chest imaging, prospective collection of medication history, and complete blood count with differential, coagulation studies, and chemistry panel.

On the morning of the bronchoscopy visit, with the participants having taken nothing by mouth for at least 8 h, EBC samples were obtained using the RTube collection system from Respiratory Research, Inc. (Charlottesville, NC), according to the manufacturer’s instructions. Consistent with American Thoracic Society recommendations (24), participants wore a nose clip and were coached to breath naturally without hyperventilation for approximately 20 min through a one-way valve into a cooled chamber that condenses and collects up to 2 ml of expired vapors, aerosols, and moisture.

Next, before any other procedures, participants provided an oral rinse specimen, as described previously (25). They then underwent bronchoscopy under moderate conscious sedation, using an orally inserted fiber-optic bronchoscope. We performed bilateral BAL in the right middle lobe and lingula, followed by collection of a posterior nasopharyngeal swab, as described in detail elsewhere (2, 25, 26), but omitting protected specimen brushing. We also created a sample called combined BAL (CBAL) fluid from an equal mix of left-lung and right-lung BAL fluid samples. We collected control samples specific to the experimental samples wherever possible: sterile saline for the oral washes, sterile saline passed as a prewash through the bronchoscope for the BAL fluid specimens, unused swabs for nasal swabs, and EBC control samples obtained by passing 1 ml of sterile double-distilled water over a fresh RTube and collecting the water. Isolation controls were generated by carrying out the DNA isolation procedure without the addition of any sample.

### DNA isolation.

All liquid samples were processed as described previously (19). Briefly, we aliquoted samples into dolphin-nose Eppendorf tubes and spun them at an rcf of 18,000 at 4°C in a microcentrifuge for 30 min to pellet the cells (bacterial and host). We then extracted DNA from the pellet using the Qiagen DNeasy kits with the addition of bead beating. Brushes or swabs were placed directly in the bead beating tubes, and we carried out the remaining steps of DNA isolation according to manufacturer protocols with minor modifications (19). DNA extraction controls were used for DNA contamination within extraction reagents. In addition to extraction controls that related to the kits used for DNA isolation for the clinical samples, extraction controls were generated from seven total separate DNA isolation kits to examine reagent contamination across multiple lots.

### Library preparation and sequencing.

We prepared DNA libraries by amplifying the V4 region of the bacterial 16S rRNA gene using a low-bacterial-biomass protocol as described previously (19) with a dual-indexing strategy (27). Sequencing was performed on a MiSeq instrument (Illumina, San Diego, CA) at the Microbial Systems Molecular Biology Laboratory at the University of Michigan. Samples were sequenced in three runs: one comprised the dilution replicates and extraction control experiments (Fig. 1), and the other two runs were composed of the clinical samples. Where physically possible, all samples and replicates from an individual were run on the same plate.

### Bacterial load quantification.

Bacterial DNA was quantified using a QX200 Droplet digital PCR system (Bio-Rad, Hercules, CA). Quantitation was performed as described previously (28).

### Sequence processing and analysis.

Fastq files were obtained, and the sequences were processed using the open source software mothur v.1.36 according to the MiSeq SOP minor alterations. We used the SILVA bacterial database for alignment and binned operational taxonomic units (OTUs) at 97% similarity. We generated taxonomies using the RDP taxonomy. After generation of the .shared file and the .cons.taxonomy file, these files were imported into R for final analysis. The R packages that we used for analysis relied heavily upon base-R, vegan (29), ComplexHeatmap (30), ggplot2 (31), dplyr (32), tidyr (33), mvabund (34), and RColorBrewer (35). Additionally, we used Prism 8 (GraphPad Software, San Diego, CA) to generate figures. Final figures were assembled in Photoshop CS5 (Adobe Inc.). Analysis of the controls (Fig. 1C and Fig. S1 to S9 in the supplemental material) indicated that three OTUs were likely contaminants. These OTUs were removed from all clinical samples to eliminate elements that could artificially make samples look more similar to one another. To ensure that removal of the OTUs did not remove all viable counts from a sample, the count totals before and after removal were compared; an average of roughly 90% (∼19,000 reads/sample) of the reads were retained. It should be noted that how to deal with backgrounds is a complex issue given the compositional nature of microbiome data and should be evaluated with care.

10.1128/mBio.00258-20.1 FIG S1 Mock community replicates. Abundances for eight samples of the Zymo Mock community (means and medians). Download FIG S1, JPG file, 0.8 MB. Copyright © 2020 Erb-Downward et al. 2020 Erb-Downward et al. This content is distributed under the terms of the Creative Commons Attribution 4.0 International license.

### Data availability.

Sequences have been deposited in the SRA under accession numbers PRJNA549253 and PRJNA552077. Code and other files related to the project can be found at https://github.com/Tetrakis/low-biomass-noise.

10.1128/mBio.00258-20.2 FIG S2 DNA isolation control 1. One of the seven separate DNA isolation controls used in this experiment. Three replicates of the isolation control, with their means and medians. Download FIG S2, JPG file, 0.7 MB. Copyright © 2020 Erb-Downward et al. 2020 Erb-Downward et al. This content is distributed under the terms of the Creative Commons Attribution 4.0 International license.

10.1128/mBio.00258-20.3 FIG S3 DNA isolation control 2. One of the seven separate DNA isolation controls used in this experiment. Three replicates of the isolation control, with their means and medians. Download FIG S3, JPG file, 0.7 MB. Copyright © 2020 Erb-Downward et al. 2020 Erb-Downward et al. This content is distributed under the terms of the Creative Commons Attribution 4.0 International license.

10.1128/mBio.00258-20.4 FIG S4 DNA isolation control 3. One of the seven separate DNA isolation controls used in this experiment. Three replicates of the isolation control, with their means and medians. Download FIG S4, JPG file, 0.7 MB. Copyright © 2020 Erb-Downward et al. 2020 Erb-Downward et al. This content is distributed under the terms of the Creative Commons Attribution 4.0 International license.

10.1128/mBio.00258-20.5 FIG S5 DNA isolation control 4. One of the seven separate DNA isolation controls used in this experiment. Three replicates of the isolation control, with their means and medians. Download FIG S5, JPG file, 0.7 MB. Copyright © 2020 Erb-Downward et al. 2020 Erb-Downward et al. This content is distributed under the terms of the Creative Commons Attribution 4.0 International license.

10.1128/mBio.00258-20.6 FIG S6 DNA isolation control 5. One of the seven separate DNA isolation controls used in this experiment. Three replicates of the isolation control, with their means and medians. Download FIG S6, JPG file, 0.7 MB. Copyright © 2020 Erb-Downward et al. 2020 Erb-Downward et al. This content is distributed under the terms of the Creative Commons Attribution 4.0 International license.

10.1128/mBio.00258-20.7 FIG S7 DNA isolation control 6. One of the seven separate DNA isolation controls used in this experiment. Three replicates of the isolation control, with their means and medians. Download FIG S7, JPG file, 0.7 MB. Copyright © 2020 Erb-Downward et al. 2020 Erb-Downward et al. This content is distributed under the terms of the Creative Commons Attribution 4.0 International license.

10.1128/mBio.00258-20.8 FIG S8 DNA isolation control 7. One of the seven separate DNA isolation controls used in this experiment. Three replicates of the isolation control, with their means and medians. Download FIG S8, JPG file, 0.7 MB. Copyright © 2020 Erb-Downward et al. 2020 Erb-Downward et al. This content is distributed under the terms of the Creative Commons Attribution 4.0 International license.

10.1128/mBio.00258-20.9 FIG S9 Mean of replicate medians of DNA isolation controls. To identify contaminants, the mean of replicate medians of the DNA isolation controls was used. Download FIG S9, JPG file, 0.1 MB. Copyright © 2020 Erb-Downward et al. 2020 Erb-Downward et al. This content is distributed under the terms of the Creative Commons Attribution 4.0 International license.

## References

[1] Bassis CM, Erb-Downward JR, Dickson RP, Freeman CM, Schmidt TM, Young VB, Beck JM, Curtis JL, Huffnagle GB 2015 Analysis of the upper respiratory tract microbiotas as the source of the lung and gastric microbiotas in healthy individuals. mBio 6:e00037. doi:10.1128/mBio.00037-15. 25736890 PMC4358017

[2] Dickson RP, Erb-Downward JR, Freeman CM, McCloskey L, Falkowski NR, Huffnagle GB, Curtis JL 2017 Bacterial topography of the healthy human lower respiratory tract. mBio 8:e02287-16. doi:10.1128/mBio.02287-16. 28196961 PMC5312084

[3] Erb-Downward JR, Thompson DL, Han MK, Freeman CM, McCloskey L, Schmidt LA, Young VB, Toews GB, Curtis JL, Sundaram B, Martinez FJ, Huffnagle GB 2011 Analysis of the lung microbiome in the “healthy” smoker and in COPD. PLoS One 6:e16384. doi:10.1371/journal.pone.0016384. 21364979 PMC3043049

[4] Hilty M, Burke C, Pedro H, Cardenas P, Bush A, Bossley C, Davies J, Ervine A, Poulter L, Pachter L, Moffatt MF, Cookson WO 2010 Disordered microbial communities in asthmatic airways. PLoS One 5:e8578. doi:10.1371/journal.pone.0008578. 20052417 PMC2798952

[5] Parnell LA, Briggs CM, Cao B, Delannoy-Bruno O, Schrieffer AE, Mysorekar IU 2017 Microbial communities in placentas from term normal pregnancy exhibit spatially variable profiles. Sci Rep 7:11200. doi:10.1038/s41598-017-11514-4. 28894161 PMC5593928

[6] Prince AL, Ma J, Kannan PS, Alvarez M, Gisslen T, Harris RA, Sweeney EL, Knox CL, Lambers DS, Jobe AH, Chougnet CA, Kallapur SG, Aagaard KM 2016 The placental membrane microbiome is altered among subjects with spontaneous preterm birth with and without chorioamnionitis. Am J Obstet Gynecol 214:627.e1–627.e16. doi:10.1016/j.ajog.2016.01.193. 26965447 PMC4909356

[7] Zheng J, Xiao X, Zhang Q, Mao L, Yu M, Xu J, Wang T 2017 The placental microbiota is altered among subjects with gestational diabetes mellitus: a pilot study. Front Physiol 8:675. doi:10.3389/fphys.2017.00675. 28932201 PMC5592210

[8] Glendinning L, Wright S, Tennant P, Gill AC, Collie D, McLachlan G 2017 Microbiota in exhaled breath condensate and the lung. Appl Environ Microbiol 83:e00515-17. doi:10.1128/AEM.00515-17. 28389539 PMC5452816

[9] Zakharkina T, Koczulla AR, Mardanova O, Hattesohl A, Bals R 2011 Detection of microorganisms in exhaled breath condensate during acute exacerbations of COPD. Respirology 16:932–938. doi:10.1111/j.1440-1843.2011.01977.x. 21470340

[10] Lauder AP, Roche AM, Sherrill-Mix S, Bailey A, Laughlin AL, Bittinger K, Leite R, Elovitz MA, Parry S, Bushman FD 2016 Comparison of placenta samples with contamination controls does not provide evidence for a distinct placenta microbiota. Microbiome 4:29. doi:10.1186/s40168-016-0172-3. 27338728 PMC4917942

[11] Leiby JS, McCormick K, Sherrill-Mix S, Clarke EL, Kessler LR, Taylor LJ, Hofstaedter CE, Roche AM, Mattei LM, Bittinger K, Elovitz MA, Leite R, Parry S, Bushman FD 2018 Lack of detection of a human placenta microbiome in samples from preterm and term deliveries. Microbiome 6:196. doi:10.1186/s40168-018-0575-4. 30376898 PMC6208038

[12] Theis KR, Romero R, Winters AD, Greenberg JM, Gomez-Lopez N, Alhousseini A, Bieda J, Maymon E, Pacora P, Fettweis JM, Buck GA, Jefferson KK, Strauss JF III, Erez O, Hassan SS 2019 Does the human placenta delivered at term have a microbiota? Results of cultivation, quantitative real-time PCR, 16S rRNA gene sequencing, and metagenomics. Am J Obstet Gynecol 220:267.e1–267.e39. doi:10.1016/j.ajog.2018.10.018. 30832984 PMC6733039

[13] St. George K, Fuschino ME, Mokhiber K, Triner W, Spivack SD 2010 Exhaled breath condensate appears to be an unsuitable specimen type for the detection of influenza viruses with nucleic acid-based methods. J Virol Methods 163:144–146. doi:10.1016/j.jviromet.2009.08.019. 19733195 PMC3730442

[14] Vogelberg C, Hirsch T, Rosen-Wolff A, Kerkmann ML, Leupold W 2003 Pseudomonas aeruginosa and Burkholderia cepacia cannot be detected by PCR in the breath condensate of patients with cystic fibrosis. Pediatr Pulmonol 36:348–352. doi:10.1002/ppul.10352. 12950050

[15] Salter SJ, Cox MJ, Turek EM, Calus ST, Cookson WO, Moffatt MF, Turner P, Parkhill J, Loman NJ, Walker AW 2014 Reagent and laboratory contamination can critically impact sequence-based microbiome analyses. BMC Biol 12:87. doi:10.1186/s12915-014-0087-z. 25387460 PMC4228153

[16] Costello M, Fleharty M, Abreu J, Farjoun Y, Ferriera S, Holmes L, Granger B, Green L, Howd T, Mason T, Vicente G, Dasilva M, Brodeur W, DeSmet T, Dodge S, Lennon NJ, Gabriel S 2018 Characterization and remediation of sample index swaps by non-redundant dual indexing on massively parallel sequencing platforms. BMC Genomics 19:332. doi:10.1186/s12864-018-4703-0. 29739332 PMC5941783

[17] MacConaill LE, Burns RT, Nag A, Coleman HA, Slevin MK, Giorda K, Light M, Lai K, Jarosz M, McNeill MS, Ducar MD, Meyerson M, Thorner AR 2018 Unique, dual-indexed sequencing adapters with UMIs effectively eliminate index cross-talk and significantly improve sensitivity of massively parallel sequencing. BMC Genomics 19:30. doi:10.1186/s12864-017-4428-5. 29310587 PMC5759201

[18] Minich JJ, Sanders JG, Amir A, Humphrey G, Gilbert JA, Knight R 2019 Quantifying and understanding well-to-well contamination in microbiome research. mSystems 4:e00186-19. doi:10.1128/mSystems.00186-19. PMC6593221 31239396

[19] Dickson RP, Erb-Downward JR, Freeman CM, Walker N, Scales BS, Beck JM, Martinez FJ, Curtis JL, Lama VN, Huffnagle GB 2014 Changes in the lung microbiome following lung transplantation Include the emergence of two distinct Pseudomonas species with distinct clinical associations. PLoS One 9:e97214. doi:10.1371/journal.pone.0097214. 24831685 PMC4022512

[20] Prosser JI 2010 Replicate or lie. Environ Microbiol 12:1806–1810. doi:10.1111/j.1462-2920.2010.02201.x. 20438583

[21] de Goffau MC, Lager S, Sovio U, Gaccioli F, Cook E, Peacock SJ, Parkhill J, Charnock-Jones DS, Smith G 2019 Human placenta has no microbiome but can contain potential pathogens. Nature 572:329–334. doi:10.1038/s41586-019-1451-5. 31367035 PMC6697540

[22] Sze MA, Schloss PD 2019 The impact of DNA polymerase and number of rounds of amplification in PCR on 16S rRNA gene sequence data. mSphere 4:e00163-19. doi:10.1128/mSphere.00163-19. 31118299 PMC6531881

[23] Akbari M, Hansen MD, Halgunset J, Skorpen F, Krokan HE 2005 Low copy number DNA template can render polymerase chain reaction error prone in a sequence-dependent manner. J Mol Diagn 7:36–39. doi:10.1016/S1525-1578(10)60006-2. 15681472 PMC1867510

[24] Horvath I, Hunt J, Barnes PJ, Alving K, Antczak A, Baraldi E, Becher G, van Beurden WJ, Corradi M, Dekhuijzen R, Dweik RA, Dwyer T, Effros R, Erzurum S, Gaston B, Gessner C, Greening A, Ho LP, Hohlfeld J, Jobsis Q, Laskowski D, Loukides S, Marlin D, Montuschi P, Olin AC, Redington AE, Reinhold P, van Rensen EL, Rubinstein I, Silkoff P, Toren K, Vass G, Vogelberg C, Wirtz H, ATS/ERS Task Force on Exhaled Breath Condensate. 2005 Exhaled breath condensate: methodological recommendations and unresolved questions. Eur Respir J 26:523–548. doi:10.1183/09031936.05.00029705. 16135737

[25] Morris A, Lung HIV Microbiome Project, Beck JM, Schloss PD, Campbell TB, Crothers K, Curtis JL, Flores SC, Fontenot AP, Ghedin E, Huang L, Jablonski K, Kleerup E, Lynch SV, Sodergren E, Twigg H, Young VB, Bassis CM, Venkataraman A, Schmidt TM, Weinstock GM 2013 Comparison of the respiratory microbiome in healthy nonsmokers and smokers. Am J Respir Crit Care Med 187:1067–1075. doi:10.1164/rccm.201210-1913OC. 23491408 PMC3734620

[26] Dickson RP, Erb-Downward JR, Freeman CM, McCloskey L, Beck JM, Huffnagle GB, Curtis JL 2015 Spatial variation in the healthy human lung microbiome and the adapted island model of lung biogeography. Annals ATS 12:821–830. doi:10.1513/AnnalsATS.201501-029OC. PMC4590020 25803243

[27] Kozich JJ, Westcott SL, Baxter NT, Highlander SK, Schloss PD 2013 Development of a dual-index sequencing strategy and curation pipeline for analyzing amplicon sequence data on the MiSeq Illumina sequencing platform. Appl Environ Microbiol 79:5112–5120. doi:10.1128/AEM.01043-13. 23793624 PMC3753973

[28] Dickson RP, Erb-Downward JR, Falkowski NR, Hunter EM, Ashley SL, Huffnagle GB 2018 The lung microbiota of healthy mice are highly variable, cluster by environment, and reflect variation in baseline lung innate immunity. Am J Respir Crit Care Med 198:497–508. doi:10.1164/rccm.201711-2180OC. 29533677 PMC6118022

[29] Oksanen J, Blanchet FG, Friendly M, Kindt R, Legendre P, McGlinn D, Minchin P, O’Hara RB, Simpson GL, Solymos P, Stevens MHH, Szoecs E, Wagner H 2018 vegan: community ecology package. R package version 2.5–3. https://CRAN.R-project.org/package=vegan.

[30] Gu Z, Eils R, Schlesner M 2016 Complex heatmaps reveal patterns and correlations in multidimensional genomic data. Bioinformatics 32:2847–2849. doi:10.1093/bioinformatics/btw313. 27207943

[31] Wickham H 2016 ggplot2: elegant graphics for data analysis. Springer, New York, NY.

[32] Wickham H, François R, Henry L, Müller K 2018 dplyr: a grammar of data manipulation. vR package version 0.7.6. https://CRAN.R-project.org/package=dplyr.

[33] Henry H, Henry L 2018 tidyr: easily tidy data with ‘spread()’ and ‘gather()’ functions. vR package version 0.8.1. https://CRAN.R-project.org/package=tidyr.

[34] Wang Y, Naumann U, Eddelbuettel D, Wilshire J, Warton D 2019 mvabund: statistical methods for analysing multivariate abundance data. vR package version 4.0.1. https://CRAN.R-project.org/package=mvabund.

[35] Neuwirth E 2014 RColorBrewer: ColorBrewer palettes. vR package version 1.1–2. https://CRAN.R-project.org/package=RColorBrewer.

